# Reprogramming cells to study vacuolar development

**DOI:** 10.3389/fpls.2013.00493

**Published:** 2013-12-03

**Authors:** Mistianne Feeney, Lorenzo Frigerio, Susanne E. Kohalmi, Yuhai Cui, Rima Menassa

**Affiliations:** ^1^Department of Biology, University of Western OntarioLondon, ON, Canada; ^2^Southern Crop Protection and Food Research Centre, Agriculture and Agri-Food CanadaLondon, ON, Canada; ^3^School of Life Sciences, University of WarwickCoventry, UK

**Keywords:** *Arabidopsis thaliana*, cellular reprogramming, developmental transition, LEAFY COTYLEDON2, lytic vacuole, protein storage vacuole, vacuole biogenesis

## Abstract

During vegetative and embryonic developmental transitions, plant cells are massively reorganized to support the activities that will take place during the subsequent developmental phase. Studying cellular and subcellular changes that occur during these short transitional periods can sometimes present challenges, especially when dealing with *Arabidopsis thaliana* embryo and seed tissues. As a complementary approach, cellular reprogramming can be used as a tool to study these cellular changes in another, more easily accessible, tissue type. To reprogram cells, genetic manipulation of particular regulatory factors that play critical roles in establishing or repressing the seed developmental program can be used to bring about a change of cell fate. During different developmental phases, vacuoles assume different functions and morphologies to respond to the changing needs of the cell. Lytic vacuoles (LVs) and protein storage vacuoles (PSVs) are the two main vacuole types found in flowering plants such as *Arabidopsis*. Although both are morphologically distinct and carry out unique functions, they also share some similar activities. As the co-existence of the two vacuole types is short-lived in plant cells, how they replace each other has been a long-standing curiosity. To study the LV to PSV transition, *LEAFY COTYLEDON2*, a key transcriptional regulator of seed development, was overexpressed in vegetative cells to activate the seed developmental program. At the cellular level, *Arabidopsis* leaf LVs were observed to convert to PSV-like organelles. This presents the opportunity for further research to elucidate the mechanism of LV to PSV transitions. Overall, this example demonstrates the potential usefulness of cellular reprogramming as a method to study cellular processes that occur during developmental transitions.

## INTRODUCTION

**FIGURE 1:**
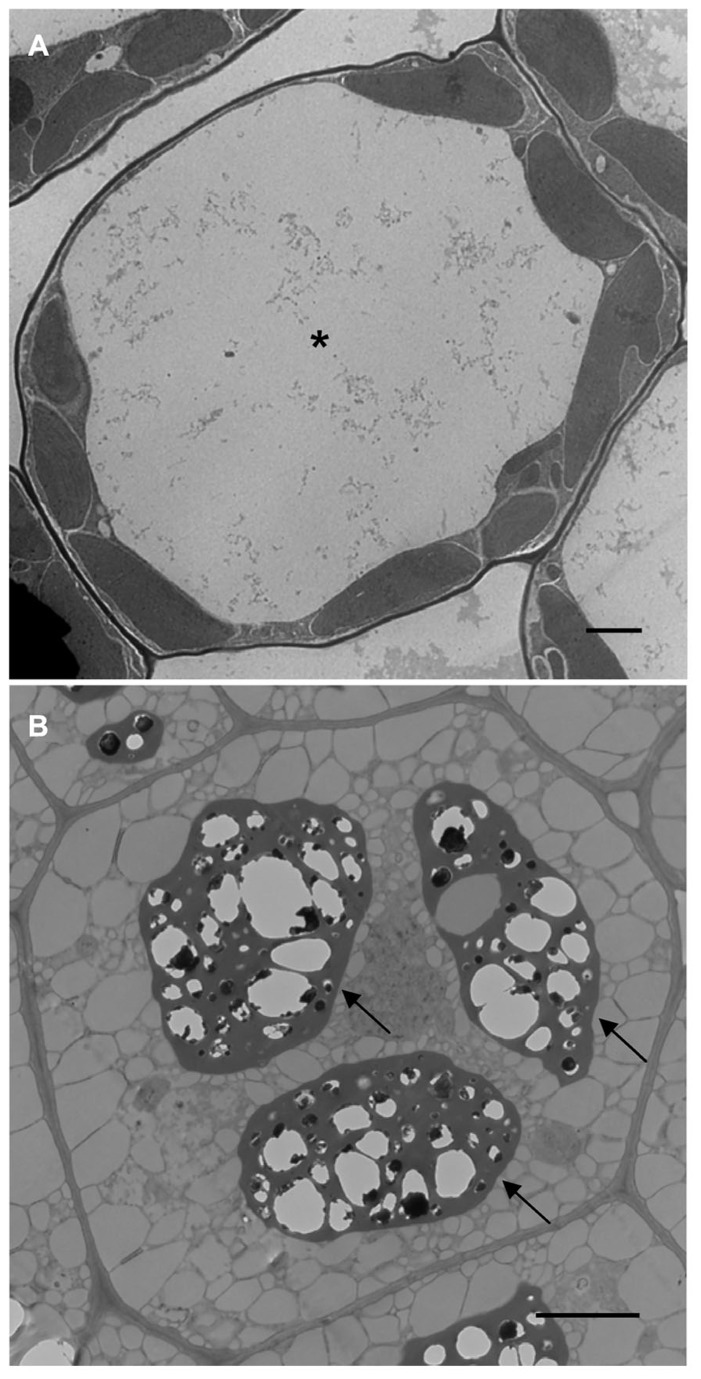
**Transmission electron microscope images of *Arabidopsis* leaf (A) and seed (B) cells.** Asterisk shows the LV and arrows point to PSVs. Bars = 2 μm.

One of the most dramatic cellular changes that occur over the lifetime of a flowering plant happens during the transition between vegetative and embryonic developmental phases. Vegetative cells are massively reorganized to support embryonic development and *vice versa* (Mansfield and Briarty, 1991, 1992, 1996). The array of organelles present within the cells remains more or less constant, however, vegetative and embryonic cells are morphologically distinct (**Figures 1A,B**). At the subcellular level, what happens to the organelles during these transitions?

Studying the cellular and subcellular changes that occur during these developmental transitions is challenging. *Arabidopsis thaliana* is the model organism of the plant community and has been adopted for genetic, physiology, molecular, and developmental biology research (Meyerowitz, 2001; Somerville and Koornneef, 2002). Thus, it is beneficial and practical to relate these areas of study to the cell biology of this plant. In *Arabidopsis*, however, it is difficult to study the cellular changes that occur during vegetative and embryonic developmental programs on account of the small seed size and technical challenges in isolating embryos from maternal tissues at early stages of embryogenesis (Girke et al., 2000; Xiang et al., 2011; Ibl and Stoger, 2012).

During different developmental stages of the plant life cycle, vacuoles assume diverse functions in response to the changing needs of the cell, and their morphology will be significantly different (Marty, 1999; Zouhar and Rojo, 2009). Due to its sheer size, the lytic vacuole (LV) is the most prominent organelle in the vegetative plant cell. Its counterpart, present in *Arabidopsis* seeds, is the protein storage vacuole (PSV) which looks nothing like the LV (**Figures 1A,B**). It is remarkable how such a large organelle (LV) can transform (Zheng and Staehelin, 2011) or be replaced (Hoh et al., 1995) by much smaller and more numerous PSVs and *vice versa*. Another intriguing issue is how the two vacuoles can have such drastically different functions; the LV is essential for water (Beebo et al., 2009) and ionic homeostasis (Isayenkov et al., 2010), plays a role in shaping vegetative cells (Rojo et al., 2001), and also acts as a cellular waste bin (Li and Vierstra, 2012). In contrast, the PSV stores protein (Scarafoni et al., 2001) and mineral reserves (Otegui et al., 2002) and therefore acts as a cellular pantry. PSVs are present as storage organelles in three of the world’s major food crops (Varshney et al., 2012); rice (Kawakatsu et al., 2010), wheat (Regvar et al., 2011), and maize (Reyes et al., 2011) and are prevalent in other important food sources such as nuts, legumes, and other cereals (Bethke et al., 1998; Hara-Nishimura et al., 1998; Robinson et al., 2005). Indeed, PSVs play an important role in supplying essential nutrients for our dietary needs. Here we discuss how cellular reprogramming can be used to learn more about LV to PSV transitions in *Arabidopsis*.

## LYTIC VACUOLE STRUCTURE AND FUNCTION

The central LV is the largest and most instantly recognizable organelle in a vegetative plant cell. It can account for up to 90% of the total cell volume (Jolliffe et al., 2005). As such, the LV squeezes the cytoplasm and other organelles between the tonoplast and plasma membrane (**Figure 1A**). LVs are present in the cells of young seedlings shortly after germination and generally exist in all cells throughout vegetative growth (Gattolin et al., 2009; Zheng and Staehelin, 2011).

The LV lumen contains water, numerous hydrolytic enzymes, and is maintained at a pH of 5.5–6 (Martinière et al., 2013). The tonoplast plays a major role in maintaining this luminal environment. The tonoplast is a selective membrane that contains a large number of channel and transport proteins that mediate the movement of organic and ionic substances between the cytoplasm and vacuole (Carter et al., 2004; Jaquinod et al., 2007; Müntz, 2007). To maintain an acidic luminal pH, vacuolar ATPase (V-ATPase) and pyrophosphatase (V-PPase) catalyze ATP-dependent proton transfer across the tonoplast. Their activity creates a proton gradient and membrane potential which energizes secondary active transport across the tonoplast (Yang et al., 2007; Krebs et al., 2010). The movement of inorganic metabolites is mediated by specific ion channels (Voelker et al., 2006) or transporters (Brini et al., 2007), while organic substances are moved by ATP-binding cassette (ABC) transporters (Shi et al., 2007; Song et al., 2010). The movement of water across the tonoplast is facilitated by water channels called aquaporins (Maurel et al., 2008). Within the vacuole lumen, numerous hydrolytic enzymes are present such as proteases, glycosidases, lipases, nucleases, and peroxidases (Carter et al., 2004).

The LV participates in diverse physical and metabolic functions that are critical for the survival of a plant. A significant role of the LV is to allow the cell to increase its size without expending too much energy. This allows lower cost cellular growth as vacuoles largely consist of water and have a low density of organic compounds to synthesize (Zouhar and Rojo, 2009). One universally important function of the LV is its role in maintaining turgor pressure which determines the rigidity of the cell and is important for growth and mechanical stability of the plant (Müntz, 2007). In addition to physical functions, LVs play important metabolic roles by storing a large variety of compounds such as toxins (Riechers et al., 2010), salts (Krebs et al., 2010), heavy metals (Song et al., 2010), pigments (Reuveni et al., 2001; Zhang et al., 2006), and defense compounds (Zhao and Dixon, 2010). The low pH and numerous hydrolytic enzymes present in the LV lumen allow it to play a fundamental role in the degradation of cytoplasmic materials from small molecules to organelles. This process involves autophagy, a conserved mechanism in eukaryotes whereby cell contents are transferred to the vacuole to be digested and recycled, typically in a non-selective manner (Bassham, 2007; Li and Vierstra, 2012). Generally, a basal level of autophagy functions constitutively for the turnover of cellular components (Wang et al., 2013). However, it can be induced to higher levels during particular developmental stages (Bassham et al., 2006) or in times of cell stress (Liu et al., 2012). Endocytic trafficking of proteins from the plasma membrane is an essential cellular transport system required for cell communication, cellular differentiation, and physiological responses to the environment (Otegui and Spitzer, 2008; Irani and Russinova, 2009; Richter et al., 2009). The LV plays a key role as the cellular endpoint where selected endocytosed proteins are sent for degradation (Otegui and Spitzer, 2008).

## PROTEIN STORAGE VACUOLE STRUCTURE AND FUNCTION

The PSV is a specialized organelle that is unique to flowering plants (Wang et al., 2012). It is found predominantly in seeds and young meristematic plant cells (Olbrich et al., 2007; Gattolin et al., 2011). In shoot and root meristem cells, the existence of PSVs is thought to be due to the persistence of seed-specific signals in the meristem (Olbrich et al., 2007). Whereas LVs typically occupy most of the cell space, PSVs are much smaller in size and range from 1.5 to 8 μm in diameter (Gillespie et al., 2005). PSVs are numerous and are usually positioned close to the center of the cell (Shimada et al., 2008; **Figure 1B**).

The PSV is a compartmentalized organelle (Jiang et al., 2001; Bolte et al., 2011; Regvar et al., 2011). Comparatively less is known about the PSV tonoplast than the LV tonoplast (Isayenkov et al., 2010) but they are known to share some similarity in their composition of proteins (Hoh et al., 1995; Jiang et al., 2001; Isayenkov et al., 2011). The pH of the PSV lumen varies between 4.9 and 5.5 (Otegui et al., 2006). The defining feature of PSVs is their ability to accumulate seed storage proteins (SSPs). In fact, the appearance of PSVs in embryonic cells coincides with storage reserve accumulation during the maturation phase of embryogenesis (Mansfield and Briarty, 1992). Lesser recognized roles of PSVs are the storage of phosphorus and minerals (Otegui et al., 2002), protective compounds such as lectins (De Hoff et al., 2009) and chitinases (Neuhaus et al., 1991), and proteolytic enzymes (Gruis et al., 2004). Like their LV counterparts, PSVs are also involved in autophagy. PSVs of wheat and maize sequester SSPs from endoplasmic reticulum (ER)-derived protein bodies (Levanony et al., 1992) or prevacuolar-like compartments (Reyes et al., 2011) by autophagic mechanisms, respectively.

## VACUOLE CONVERSION DURING EMBRYONIC AND VEGETATIVE GROWTH AND DEVELOPMENT

### MULTIPLE VACUOLES IN PLANT CELLS

As discussed, seed plants have two principal, functionally distinct vacuole types, LVs and PSVs (Becker, 2007; Ibl and Stoger, 2012). This has raised questions about whether the two vacuoles co-exist in cells. To address this question, the two vacuole types must be distinguished. LVs and PSVs can be differentiated by the presence of tonoplast intrinsic protein (TIP) isoforms (Jauh et al., 1999; Gattolin et al., 2010). TIPs are aquaporins that are specifically localized to tonoplasts (Maurel et al., 2009). *Arabidopsis* has 10 TIP isoforms and two of these are used to discriminate between LVs and PSVs, TIP1;1 and TIP3;1, respectively (Johanson et al., 2001; Gattolin et al., 2010). In addition, a small number of soluble proteins have been shown to reside exclusively within the lumen of each vacuole type and thus also serve as markers to discriminate between both vacuole types. For example, the cysteine protease aleurain is targeted to the LV (Ahmed et al., 2000), and phaseolin and 2S albumin, the major SSPs of common bean and *Arabidopsis*, respectively, are targeted to PSVs (Frigerio et al., 1998; Miao et al., 2008). LVs and PSVs were originally proposed to co-exist in plant cells based on TIP and soluble protein localization studies undertaken in the 1990s (Paris et al., 1996; Park et al., 2004). This idea was attractive because it provided a convenient explanation for the diverse sorting signals and routes that proteins follow to arrive at the two vacuole types (Vitale and Raikhel, 1999; Vitale and Hinz, 2005; Frigerio et al., 2008; Rojo and Denecke, 2008). This theory was subsequently challenged as more insight was gained by studying TIP expression patterns. *TIP3;1* and *TIP1;1 *expression were discovered to be tissue- and development-specific rather than organelle-specific (Hunter et al., 2007; Olbrich et al., 2007). Recently, a detailed map of TIP expression patterns in *Arabidopsis* has been produced to better understand the distribution pattern of these vacuolar markers (Gattolin et al., 2009, 2010, 2011). However, some differences in TIP expression patterns have been reported (Bolte et al., 2011). The current general view is that most cells contain one vacuolar type. However, both vacuoles have been shown to co-exist in some cells but their co-existence is usually short-lived (Hoh et al., 1995; Frigerio et al., 2008; Zheng and Staehelin, 2011).

### BIOGENESIS OF VACUOLES DURING DEVELOPMENTAL TRANSITIONS

#### LV to PSV Transition

During *Arabidopsis* embryogenesis, a large LV forms in the fertilized zygote. The zygote then divides to produce a vacuolated basal cell (which will form the suspensor) and a non-vacuolated apical cell (which will give rise to the embryo; De Smet et al., 2010). As the embryo continues to divide, LVs develop in all cells (Zouhar and Rojo, 2009). During the maturation phase of embryogenesis, LVs are replaced by PSVs which will accumulate SSPs and mineral reserves (Mansfield and Briarty, 1991).

#### PSV to LV Transition

During germination, storage reserves are mobilized to provide nutrients and energy for the growing embryo (Pritchard et al., 2002; Tan-Wilson and Wilson, 2012). As protein and mineral reserves are released, PSVs are replaced by LVs. The LV subsequently increases in volume to create turgor and support cell growth and expansion (Mansfield and Briarty, 1996). In *Arabidopsis*, it takes approximately 3.5 d for the PSV to LV transition to occur (Hunter et al., 2007). PSV to LV transitions are also observed in root cells. PSVs are present in the radicle as it emerges from the seed coat. As the root elongates, meristematic regions of the root tip retain PSVs while PSVs transition to LVs in the distal regions of elongating roots (Olbrich et al., 2007; Gattolin et al., 2011; Zheng and Staehelin, 2011).

How do such morphologically distinct vacuoles replace each other in the cell during vegetative and embryonic transitions? We envisage two possible scenarios: either a new vacuolar type arises by remodeling of the pre-existing vacuole, or a new vacuole is formed *de novo* and very rapidly supersedes the existing one.

#### Vacuole Remodeling

One means by which different vacuole types may replace each other is by remodeling or reprogramming the vacuole that is already present in the cell. Several studies support this hypothesis. During *Arabidopsis* germination and seedling development, Mansfield and Briarty (1996) observed multiple PSVs fusing to form a LV after the mobilization of most protein reserves. Olbrich et al. (2007) observed the formation of a single vacuole in barley and pea root tip cells. Close to the root tip, cells contain PSVs with TIP3;1 in their tonoplast. As root cells differentiate, the enlarging vacuole becomes a PSV–LV hybrid as indicated by the presence of both TIP3;1 and TIP1;1 and storage proteins in the lumen. The hybrid vacuole then gradually differentiates into a LV with increasing amounts of TIP1;1 and decreasing amounts of TIP3;1 in the tonoplast. Similarly, Zheng and Staehelin (2011) observed that PSVs in tobacco root tips were transformed into LVs. This PSV to LV transition involves unique, highly tissue-specific spatial and temporal changes in vacuole architecture. In addition, within some cell types, the transformation was shown to involve autophagosome formation and engulfment by the developing LV (Zheng and Staehelin, 2011).

#### *De novo* Vacuole Formation

An alternative hypothesis to explain how different vacuole types arise involves the independent generation of a vacuole within a cell that already has a pre-existing vacuole. A key study to support this theory was conducted by Hoh et al. (1995) who investigated the formation of PSVs in pea cotyledons during seed development. The authors observed the development of a tubular PSV structure which overtook the pre-existing LV. A second example backing this hypothesis is the demonstration that vacuoles can be regenerated from evacuolated protoplasts (Hörtensteiner et al., 1992; Di Sansebastiano et al., 2001). Using autophagy inhibitors, an autophagy-like mechanism was demonstrated to be involved in vacuole biogenesis of evacuolated tobacco protoplasts (Yano et al., 2007). However, the mechanism is distinct from conserved autophagy pathways (Bassham, 2007). Most recently, Viotti et al. (2013) also demonstrated the formation of autophagosome-like structures which give rise to LVs in *Arabidopsis* root tip cells but showed that these structures were not formed by the core autophagy machinery.

If the *de novo* theory holds true, then where does the membrane for a new vacuole originate? If an autophagy-like mechanism is involved in biogenesis, perhaps the tonoplast is generated by a process similar to autophagosome formation (Li and Vierstra, 2012). In autophagosome formation, the first step is the formation of an isolation membrane which occurs in the cytoplasm close to the vacuole. This process involves the recruitment of several autophagy-related (ATG) proteins which assemble in a coordinated manner to form a cup-shaped membrane structure that elongates and eventually engulfs material to be transported to the vacuole (Mizushima, 2007; Nakatogawa et al., 2009). Mutants defective in vacuole formation have been identified (Zouhar and Rojo, 2009). Most mutations affect factors involved in membrane fusion. An essential gene involved in vacuole biogenesis has been identified as *VACUOLELESS1* (*VCL1*) through a mutant screen (Rojo et al., 2001). Loss-of-function *vcl1* embryos were unable to form vacuoles. Mutants accumulated large numbers of autophagosomes which were unable to fuse to form the vacuole but instead would fuse with the plasma membrane and deliver their vacuolar contents to the apoplasm. Thus, *VCL1* is proposed to be involved in regulating the fusion of autophagosomes to form a LV (Zouhar and Rojo, 2009). Tonoplasts have also been proposed to originate from the ER or the Golgi apparatus (Marty, 1978; Robinson and Hinz, 1997; Staehelin, 1997; Neuhaus and Rogers, 1998). Using mutants and pharmacological inhibitors which affect the biosynthetic secretory pathway, Viotti et al. (2013) demonstrated that tonoplast proteins and lipids were derived from the ER and were delivered directly, via a Golgi-independent route, to form the LV tonoplast.

## REPROGRAMMING CELLS TO STUDY ORGANELLE DYNAMICS: VACUOLES

As an alternative to studying PSV formation in developing seeds, we asked if it was possible to induce the formation of PSVs in vegetative cells. Cues prompting vegetative cells to switch to PSV formation are not well understood. Despite the fact that SSPs are the major storage reserves that accumulate in PSVs, their forced synthesis in vegetative tissues has not been demonstrated to promote PSV formation. Constitutive expression of phaseolin in alfalfa vegetative tissues did not result in a significant accumulation of the protein in cells of non-seed organs (Bagga et al., 1992). Further, phaseolin was shown to accumulate and be degraded in the LV and, in part, secreted upon overexpression in tobacco (Frigerio et al., 1998). In transgenic *Arabidopsis* plants overexpressing a chimeric 2S albumin gene, novel precursor-accumulating (PAC)-like vesicles were induced to form in leaves (Hayashi et al., 1999). Within a plant, PSVs are abundant in seed tissues and are also observed in meristematic cells in vegetative tissues (Olbrich et al., 2007). Thus it seems that for PSVs to exist, cells must be programmed to be in an embryonic state.

Genetic research has uncovered a number of genes that play critical roles in establishing or repressing embryonic cell fate (Braybrook and Harada, 2008; Zhang and Ogas, 2009; Jia et al., 2013b). Several transcription factors such as *LEAFY COTYLEDON1* (*LEC1*; Lotan et al., 1998), *LEC2* (Stone et al., 2001), *FUSCA3* (*FUS3*; Gazzarrini et al., 2004), *BABYBOOM* (*BBM*; Boutilier et al., 2002), *WUSCHEL* (*WUS*; Zuo et al., 2002), *EMBRYOMAKER* (*EMK*; Tsuwamoto et al., 2010), and *MYB118* (Wang et al., 2009) act during embryogenesis to promote seed developmental programs. In contrast, negative seed regulators such as *PICKLE* (*PKL*; Ogas et al., 1999), *POLYCOMB* *REPRESSIVE* *COMPLEX 2* (*PRC2*; Bouyer et al., 2011), *SET* *DOMAIN* *GROUP 8* (*SDG8*; Tang et al., 2012b), *BRAHMA* (*BRM*; Tang et al., 2008), *VP1/ABSCISIC ACID INSENSITIVE 3-LIKE *(*VAL*) genes (Suzuki et al., 2007) and microRNA166 (miR166; Tang et al., 2012a) are responsible for suppressing the seed program in vegetative tissues. Thus, overexpression or downregulation of these positive and negative seed regulators, respectively, will induce a seed-specific developmental program causing vegetative tissues to exhibit embryonic characteristics. This scenario presents an opportunity to study the cellular and subcellular changes that take place during this developmental transition.

The maturation phase of embryogenesis represents an exciting window of time to study organelle dynamics. One of the most distinctive activities that take place is a high level of storage reserve accumulation (Mansfield and Briarty, 1992). In *Arabidopsis* seeds, lipids and proteins usually accumulate up to 30–40% each of the seed dry weight. Lipids are stored in oil bodies which originate from the ER membrane (Hsieh and Huang, 2004) and PSVs arise to accumulate SSPs as discussed (Mansfield and Briarty, 1992). The events that take place during the maturation phase are controlled by the complex seed regulatory network introduced above (Santos-Mendoza et al., 2008). A key part of this control is achieved through the activities of a small number of transcriptional regulators; *LEC1*, *LEC2*, *FUS3,* and *ABSCISIC ACID INSENSITIVE3* (*ABI3*; Zhang and Ogas, 2009). Genetic studies showed that vegetative tissues overexpressing these transcription factors would begin to exhibit seed traits (Gazzarrini et al., 2004; Kagaya et al., 2005; Stone et al., 2008; Junker et al., 2012).

**FIGURE 2:**
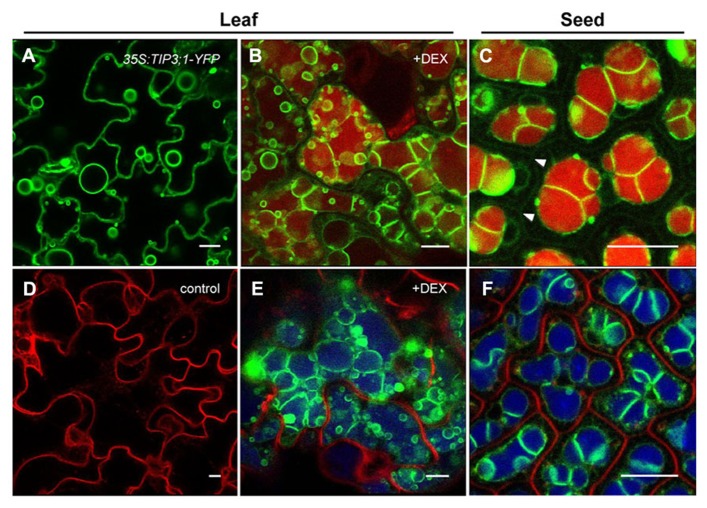
**Formation of PSV-like organelles in leaves overexpressing *LEC2*.** Leaf epidermal cells from *35S:LEC2-GR *plants co-expressing *TIP3;1:TIP3;1-YFP* and *TIP1;1:TIP1;1-RFP* (*LEC2/TIP3-YFP/TIP1-RFP*; Feeney et al., 2013) incubated on DEX to induce *LEC2* expression **(B,E)** are compared to *LEC2/TIP3-YFP/TIP1-RFP* plants grown without DEX **(D)** or leaves of seedlings constitutively expressing *35S:TIP3;1-YFP* **(A)**. Leaf cell morphology is compared with cotyledonary cells of *LEC2/TIP3-YFP/TIP1-RFP* embryos **(C,F)**. Note that *TIP1;1:TIP1;1-RFP* is not expressed in seeds and is not detected on LV tonoplasts in older vegetative tissues (**D**; Feeney et al., 2013). Green color: TIP3;1-YFP. Vacuolar lumina are highlighted with neutral red **(B,C)**. In bottom images **(D–F)** red color is FM4-64 to label the plasma membrane and blue color is autofluorescence. Arrowheads in **(C)** indicate the plasma membrane. Bars = 10 μm.

A wealth of genetic knowledge has been gathered on *LEC2* activities (Stone et al., 2001, 2008; Kroj et al., 2003; Santos Mendoza et al., 2005; Braybrook et al., 2006; To et al., 2006; Baud et al., 2007). To learn more about the cellular changes that occur during the vegetative to embryonic transition, a dexamethasone (DEX)-inducible *LEC2-GR* expression system was exploited in *Arabidopsis* (Feeney et al., 2013). The overexpression of *LEC2* triggers massive cellular reorganization in leaves and causes these vegetative organs to exhibit embryonic characteristics. Among the many cellular and subcellular changes, the replacement of LVs with PSV-like organelles was most notable. In these leaf cells, the large LV is replaced by smaller and more numerous vacuoles that contain SSP aggregates. Upon further investigation using immunogold labeling with tonoplast and luminal markers, it was established that the small vacuoles had the features of developing PSVs (Feeney et al., 2013). Indeed, the presence of the PSV-specific TIP3;1 protein on the tonoplast (Gattolin et al., 2011) and accumulation of SSPs within the lumen of the small vacuoles indicates that the leaf vacuoles assumed a storage role (Jauh et al., 1999; Hunter et al., 2007; Olbrich et al., 2007). Furthermore, confocal analysis revealed a unique embryo-like vacuolar morphology (**Figures 2B,E**). To visualize the tonoplast of these developing leaf PSVs, *35S:LEC2-GR* plants co-expressing *TIP3;1-YFP* under its native promoter was generated. The native *TIP3;1* promoter is developmentally regulated and thus the *TIP3;1-YFP* fusion is specifically expressed in seed tissues and accumulated on PSV tonoplasts (Hunter et al., 2007). As DEX-induced plants overexpressing *LEC2* began to acquire embryogenic characteristics (Feeney et al., 2013), TIP3;1-YFP became detectable on the tonoplast of leaf cells, indicating that vacuoles were PSVs (**Figures 2B,E**). Highly fluorescent TIP3;1-YFP-labeled tonoplast folds and bulbs appeared (Saito et al., 2002, 2011). These are characteristic vacuolar morphologies of young cells (**Figure 2A**). However, the tonoplast also retained the characteristic configuration of a large LV lining the periphery of the cell. To highlight the vacuole lumen, tissues were stained with neutral red and revealed that the lumen appears to occupy the entire leaf cell (**Figure 2B**) unlike seed PSVs (**Figure 2C**). In addition, vacuolar lumina began to exhibit autofluorescence (**Figure 2E**), which was not observed in uninduced leaves (**Figure 2D**). Autofluorescent vacuole lumina are usually observed in seed PSVs (**Figure 2F**; Fuji et al., 2007; Hunter et al., 2007; Bolte et al., 2011). Therefore, leaf vacuole tonoplasts were extensively remodeled but their lumina remained large and filled the entire cell (**Figures 2B,E**). These results suggest that in response to DEX-inducible *LEC2* overexpression, leaf LVs are replaced by PSV-like organelles that bear a resemblance to both LVs and PSVs. Therefore, it appears that as the *Arabidopsis* leaf LV is replaced by a PSV, the LV tonoplast remodels before being replaced by smaller-sized PSVs (Feeney et al., 2013). While these observations seem to point toward a remodeling of existing LVs into PSVs, they do not rule out the possibility of *de novo* biogenesis of PSVs, which may overlap with LV remodeling. The next question that will need to be addressed is therefore how exactly PSVs replace LVs during *LEC2*-induced leaf cell reprogramming.

## A CHALLENGE OF CELLULAR REPROGRAMMING: KNOW YOUR GENETIC REGULATOR

A number of genetic factors have been revealed to promote cellular reprogramming and some are highlighted above. To bring about cellular changes, many of these factors are involved in complex genetic, biochemical, and physiological interactions (Gazzarrini et al., 2004; Gutierrez et al., 2007; Le et al., 2010; Xiang et al., 2011). These factors may act in a hierarchical order (Kagaya et al., 2005; To et al., 2006) and may become active at slightly different developmental times (Le et al., 2010; Willmann et al., 2011). Thus, while many of the factors display a redundancy in promoting cellular reprogramming, they may also display unique activities that affect distinct cellular processes (Baud et al., 2007; Jia et al., 2013a). These aspects should be taken into consideration when choosing a reprogramming system to study a particular cellular process. In the case of vacuoles, several factors may cause vacuolar transitions in vegetative tissues. We have demonstrated that *LEC2* overexpression causes LVs to transition to PSVs and results from overexpression studies with *LEC1* (Junker et al., 2012) and *FUS3* (Gazzarrini et al., 2004) are suggestive that these transcription factors can also bring about a change in vacuole type.

## CONCLUSION

Cellular reprogramming may be a useful means of allowing the study of cellular processes that take place during the short transitional period between two developmental programs. Several genes have been discovered that control embryonic cell identity by establishing or repressing the seed developmental program (Braybrook and Harada, 2008; Zhang and Ogas, 2009; Jia et al., 2013b). In the example presented in this review, overexpression of *LEC2* was used to activate the seed developmental program in *Arabidopsis* leaves (Santos Mendoza et al., 2005; Stone et al., 2008). This system could then be used to study the cellular and subcellular changes that ensue during the vegetative to embryonic transition (Feeney et al., 2013). The observation that LVs were replaced by PSV-like organelles in leaves presents an opportunity to elucidate the mechanism of LV to PSV transitions in *Arabidopsis*. Overall, we foresee that the major advantage of cellular reprogramming in vegetative tissues is to provide a convenient and complementary system in which to study cellular processes that normally occur during developmental transitions in developing seeds–tissues that are technically challenging to work with.

## Conflict of Interest Statement

The authors declare that the research was conducted in the absence of any commercial or financial relationships that could be construed as a potential conflict of interest.
